# Safety and tolerability of oral vorolanib for neovascular (wet) age-related macular degeneration: a phase I, open-label study

**DOI:** 10.1038/s41433-023-02496-x

**Published:** 2023-04-11

**Authors:** Yunxia Gao, Fang Lu, Xiaoxin Li, Hong Dai, Kun Liu, Xiaoling Liu, Zuhua Sun, Jin Xiang, Lieming Ding, Chris Liang, Yang Wang, Zhilin Shen, Ming Zhang

**Affiliations:** 1 Department of Ophthalmology, West China Hospital, Sichuan University, Chengdu, China; 2 Department of Ophthalmology, People Eye Center of People’s Hospital, Peking University, Beijing, China; 3 Department of Ophthalmology, Beijing Hospital, Beijing, China; 4 Department of Ophthalmology, First People’s Hospital Affiliated with Shanghai Jiao Tong University, Shanghai, China; 5 Department of Retina, School of Ophthalmology & Optometry and Eye Hospital, Wenzhou Medical University, Wenzhou, China; 6 Clinical Trial Center, West China Hospital, Sichuan University, Chengdu, China; 7 Betta Pharmaceutical Co. Ltd, Hangzhou, China; 8 X-covery Holdings, Palm Beach Gardens, FL USA

**Keywords:** Outcomes research, Pharmaceutics

## Abstract

**Objective:**

To evaluate the efficacy and safety of oral vorolanib for the treatment of neovascular (wet) age-related macular degeneration (nAMD).

**Methods:**

In the dose escalation, participants received ascending doses of oral vorolanib (25–100 mg daily). In the dose expansion, participants received recommended doses (25 and 50 mg daily).

**Results:**

Between March 15, 2015, and January 23, 2019, 41 participants were enrolled in 6 centres in China. At the data cut-off (November 14, 2019), two dose-limiting toxicities (DLTs) were observed during dose escalation (one in the 75 mg cohort and one in the 100 mg cohort). The maximum tolerated dose was not reached. Treatment-related adverse events (TRAEs) occurred in 33 (80.5%) participants, and grade 3 or higher TRAEs occurred in 12 (29.3%) participants. No fatal TRAEs were observed. Increases in the mean best-corrected visual acuity (BCVA) from baseline to Day 360 of +7.7 letters (range, −5–29; *n* = 41) were observed in participants who were administered vorolanib. Corresponding reductions in mean central subfield thickness (CST) and choroidal neovascularization (CNV) area at Day 360 were observed in these three groups.

**Conclusions:**

Oral administration of vorolanib improved visual outcomes in participants with nAMD with manageable systemic safety profiles.

## Introduction

Age-related macular degeneration (AMD) is the leading cause of irreversible blindness in the elderly population [1]. The number of people with AMD was estimated to be 196 million in 2020 and will reach 288 million in 2040 globally [2]. Wet or neovascular AMD (nAMD) is characterised by choroidal neovascularization (CNV), resulting in macular haemorrhage, effusion and fibrosis [3]. CNV only represents 10–15% of AMD diagnoses; however, it constitutes 90% of cases of severe vision loss caused by AMD [4].

There has been a paradigm shift in treatments of nAMD over the past few decades. The prevalence of AMD-caused blindness has decreased, which may be attributed to anti-vascular endothelial growth factor (VEGF) treatments. Recently, intravitreal anti-VEGF drugs (e.g., bevacizumab, ranibizumab, and aflibercept) have become the standard treatment. Additionally, brolucizumab, a novel intravitreal agent, was approved in 2019 [5]. Despite their present efficacy, anti-VEGF agents also have several issues. Participants may require a high injection frequency during years of treatment, leading to a high treatment burden. In addition, compared with participants in clinical trials, real-world participants showed worse visual outcomes, possibly due to poor compliance [6, 7]. Moreover, the risk of developing retinal scarring and geographic atrophy was increased after 2–5 years of treatment [8]. Several complications have also been observed, including vitreous and subconjunctival haemorrhage, subfoveal effusion, elevated intraocular pressure, and ocular inflammation [9–12]. In addition to VEGF, platelet-derived growth factor (PDGF) is also thought to be associated with neovascularization in AMD [13]. Furthermore, E10030, a PDGF inhibitor, in combination with ranibizumab was well tolerated with preliminary efficacy in participants with nAMD [14].

Vorolanib (X-82, CM082) is a potent oral VEGF receptor (VEGFR) and PDGF receptor (PDGFR) inhibitor, suggesting a more effective inhibition than anti-VEGF injections alone. In addition, oral vorolanib is more convenient than intravitreal injection. Vorolanib exhibited highly antiangiogenic effects in human umbilical vein endothelial cells stimulated with rHuVEGF165 and markedly inhibited retinal neovascularization and avascular area in the retina of oxygen-induced retinopathy mice [15]. Furthermore, oral administration of vorolanib reduced the CNV lesion area and pathological neovascularization in a CNV rat model [16]. Moreover, a phase I study in the US has been completed with improved visual acuity [17]. Therefore, we aimed to explore the safety and pharmacokinetics (PK) as well as preliminary efficacy of oral vorolanib in participants with nAMD.

## Methods

### Study design and participants

This phase I, open-label study consisting of dose escalation and dose expansion was conducted at 6 Chinese sites (NCT02452385 and CTR20150152). This study was approved by the local institutional review board at each participating site and conducted in accordance with the Declaration of Helsinki. All participants provided written informed consent.

Eligible participants were aged 50–80 years whose study eyes showed active CNV secondary to AMD confirmed by fluorescein angiography (FA) and/or optical coherence tomography (OCT) with Early Treatment of Diabetic Retinopathy Study (ETDRS) best-corrected visual acuity (BCVA) between 20/32 and 20/400 ETDRS letters. Eligible participants were also treatment-naïve or had discontinued previous anti-VEGF therapy for over 3 months.

### Study treatment

The schedule of procedures is shown in Table S1 in the Supplementary Material. In the dose escalation, participants in each group were assigned to receive ascending doses of vorolanib (25, 50, 75 and 100 mg daily). The dose escalated to the next level in the absence of more than two of eight patients with dose-limiting toxicity (DLT), which was defined as a drug-related safety event during the first cycle (30 days) of treatment that was severe enough to require removal of the participant from the study. The maximum tolerated dose (MTD) was defined as the maximum dose at which no more than two of eight DLTs occurred in the group.

Dose reduction was not allowed during the first month, although the drug was discontinued when participants had grade ≥3 adverse events without significant decline or recovery to baseline after more than 7 days of treatment. During the expansion and extension phases, if the absolute neutrophil count was <0.5 × 10^9^/L or the platelet count was <50 × 10^9^/L, which were related to the study drug, the drug was dose interrupted until the absolute neutrophil count returned to ≥1.0 × 10^9^/L or the baseline level or the platelet count returned to ≥75 × 10^9^/L or the baseline level within 14 days; otherwise, the drug was discontinued. In addition, the drug was discontinued when SAEs/Grade ≥3 drug-related adverse events lasted more than 14 days.

No rescue treatments were permitted in this study, given the aim of evaluating extended dosing with vorolanib. However, patients could be withdrawn from the study if they were deemed to require rescue therapy.

### Outcomes

The primary objective of this study was to assess the safety and tolerability of oral vorolanib in participants with nAMD by assessing the incidence of adverse events. The secondary objectives were the pharmacokinetics (PK) in Chinese participants with nAMD and the change from baseline in mean BCVA based on ETDRS.

### Safety assessments

Safety outcomes were monitored closely during the trial, including ophthalmic examination, DLTs, systemic and ocular adverse events (AEs), serious AEs, treatment-related AEs (TRAEs), and laboratory test results. AEs were determined by the investigator per the Common Terminology Criteria for Adverse Events version 4.0.

### PK analysis

In the initial PK evaluation after a single dose, blood samples from participants in the 25 mg group were collected predose (−0.5 h) and at 0.5, 1, 2, 3, 4, 6, 8, 12, 16, 24, 36, and 48 h post-dose; blood samples from participants in the 50 mg and 75 mg groups were collected predose (−0.5 h) and at 0.5, 1, 2, 3, 4, 6, 8, 12, 16, 24, 36, 48, and 72 h post-dose. During continuous dosing, blood in the 50 mg group was obtained on Day 7, Day 14 and Day 21 predose and on Day 30 predose and at 0.5, 1, 2, 3, 4, 6, 8, 12, 16 and 24 h post-dose, while blood in the 75 mg group was obtained on Day 7, Day 14 and Day 21 predose and on Day 30 predose and at 0.5, 1, 2, 3, 4, 6, 8, 12, 16, 24, 36, 48, and 72 h post-dose. The multiple dose data for 25 mg were not collected.

All participants moved from single dosing to multiple daily dosing. Participants in the 25 mg group received a single dose on Day 1 but did not receive vorolanib on Day 2, followed by multiple daily doses on Day 3; participants in the 50 mg, 75 mg or 100 mg group received a single dose on Day 1 but did not receive vorolanib on Day 2 and Day 3, followed by multiple daily doses on Day 4.

### Efficacy assessments

The efficacy outcomes included changes from baseline in mean BCVA based on ETDRS, central subfield thickness (CST), and CNV characteristics. CST was confirmed by a Stratus OCT (Heidelberg Engineering, Heidelberg, Germany) at screening, Day 14, and Day 30; then monthly for the next 6 months; and then every 3 months until the end of treatment. FA was performed by a Heidelberg retina tomograph (Heidelberg Engineering) with an Ophthavision Imaging System (MRP Group, Boston, MA) capture station at the screening, Day 90, Day 180, Day 270 and Day 360 visits.

### Statistical analysis

Because this was a phase I study, no power calculation was performed to determine sample size. All participants who had received at least one dose of vorolanib were included in the safety assessment. All participants who completed the course of vorolanib treatment were included for efficacy assessment. All participants who had received at least one dose of vorolanib and who had PK data were included in the PK analysis. Descriptive statistics were used for analyses. For quantitative data, the means ± standard deviations were selected. All statistical analyses were performed with SAS software, v. 9.4 (SAS Institute Inc., Cary, NC, USA).

## Results

### Baseline characteristics

Seventy-two participants were screened, of whom 41 were eligible. All 41 participants with nAMD were enrolled in four cohorts (8 participants each for 25, 50 and 75 mg; 1 for 100 mg) and an expansion cohort (8 participants each for 25 and 50 mg), consisting of 26 (63.4%) men and 15 (36.6%) women. The mean age was 67.6 years (range, 52–86). The first participant was enrolled on March 15, 2015, and 14 participants completed the 1-year core study. At baseline, 48.8% (20/41) of participants were treatment-naïve, and 51.2% (21/41) had received previous therapy. The mean BCVA at baseline was 47.6 ETDRS letters and seemed to be better in the 50 mg cohort than in the other cohorts. Similarly, the mean CNV area and CST were also better in the 50 mg cohort than in the other cohorts (Table 1).

**Table 1:** Baseline characteristics.

	25 mg (*N* = 16)	50 mg (*N* = 16)	75 mg (*N* = 8)	100 mg (*N* = 1)	All (*N* = 41)
Age (years)	69.6 (8.5)	68.0 (6.0)	63.1 (9.8)	66.0 (NA)	67.6 (7.9)
Male sex	10 (62.5%)	8 (50.0%)	7 (87.5%)	1 (100.0%)	26 (63.4%)
Duration of AMD (months)	18.5 (18.0)	20.5 (22.4)	7.1 (11.8)	12.0 (NA)	16.9 (19.0)
BCVA (letters)	43.6 (18.0)	54.1 (14.9)	45.6 (16.9)	33.0 (NA)	47.6 (16.9)
CST (μm)	455.1 (175.4)	423.7 (182.9)	442.3 (90.3)	455.0 (NA)	441.0 (161.1)
Anti-VEGF history					
Yes	9 (56.3%)	9 (56.3%)	2 (25.0%)	1 (100.0%)	21 (51.2%)
No	7 (43.7%)	7 (43.7%)	6 (75.0%)	0	20 (48.8%)

Data presented are n (%) or mean (SD).

*AMD* age-related macular degeneration, *BCVA* best-corrected visual acuity, *CST* central subfield thickness.

### Safety and tolerability

One DLT in the 75 mg cohort and one in the 100 mg cohort were observed during the dose escalation phase, and an additional 8 participants were expanded in the 25 mg and 50 mg cohorts, respectively.

A total of 39 (95.1%) participants had at least one AE, of which 33 (80.5%) had TRAEs. TRAEs of grade 3 or worse were reported in 12 (29.3%) participants. The most common TRAEs of all participants are shown in Table 2. Ocular AEs were observed in eight (19.5%) participants, but no ocular TRAEs occurred. The most commonly reported nonocular TRAE was elevated alanine aminotransferase (41.5%). No fatal TRAEs were reported in either cohort.

**Table 2:** Summary of treatment-related adverse events.

	25 mg (*N* = 16)	50 mg (*N* = 16)	75 mg (*N* = 8)	100 mg (*N* = 1)
Elevated ALT	3 (18.8%)	9 (56.3%)	5 (62.5%)	0
Elevated AST	3 (18.8%)	8 (50.0%)	4 (50.0%)	0
Elevated blood pressure	4 (25.0%)	6 (37.5%)	3 (37.5%)	0
Leucopenia	1 (6.3%)	3 (18.8%)	3 (37.5%)	0
QT interval prolongation	2 (12.5%)	4 (25.0%)	1 (12.5%)	0
Thrombocytopenia	2 (12.5%)	1 (6.3%)	3 (37.5%)	0
Neutropenia	1 (6.3%)	3 (18.8%)	2 (25.0%)	0
Hair colour changes	0	4 (25.0%)	2 (25.0%)	0

Data are number of participants (%). Treatment-related adverse events reported in ≥10% of all participants.

*ALT* alanine aminotransferase, *AST* aspartate transaminase.

Treatment-related serious AEs occurred in three (7.3%) participants: one 78-year-old participant (25 mg cohort) with grade 2 increased alanine aminotransferase and aspartate aminotransferase, one 60-year-old participant (25 mg cohort) with grade 3 increased alanine aminotransferase and aspartate aminotransferase, and one 67-year-old participant (100 mg cohort) with grade 3 increased alanine aminotransferase and aspartate aminotransferase. All participants recovered after symptomatic treatment.

### PK profile

In this study, 24 participants in the 25 mg cohort (*n* = 8), 50 mg cohort (*n* = 8) and 75 mg cohort (*n* = 8) after single dosing were included for PK. The maximum blood concentration and area under the curve of vorolanib increased linearly in a dose-dependent manner for doses of 25–50 mg after a single dose. The t_1/2_ for vorolanib was approximately 5.9–10.7 h. The detailed PK profiles for single dosing in the 25 mg group, 50 mg group and 75 mg group and multiple dosing in the 50 mg group and 75 mg group are shown in Table 3.

**Table 3:** Pharmacokinetic parameters of vorolanib after single administration and multiple dosing.

	25 mg	50 mg	75 mg
Single dose	*n* = 8	*n* = 8	*n* = 8
AUC_0-24h_, h*ng/mL	1007.4 (41.4)	1726.4 (35.1)	1704.3 (27.4)
C_max_, ng/mL	140.4 (38.8)	201.4 (37.1)	236.7 (21.7)
T_max_, h	3.03 (2.00–8.00)	6.00 (2.02–8.00)	4.00 (3.00–6.00)
t_1/2_, h	5.9 (33.3)	10.7 (90.5)	10.3 (103.3)
Multiple doses	*n* **=** 0	*n* = 8	*n* = 8
AUC_ss_, h*ng/mL	–	2178.8 (38.9)	1949.6 (34.0)
C_max,ss_, ng/mL	–	245.7 (34.6)	268.7 (35.5)
T_max,ss_, h	–	5.03 (4.00–8.00)	4.00 (2.93–6.00)
R_ac_	–	1.30 (31.3)	1.20 (36.2)
CL_ss_/F, L/h	–	26.2 (40.1)	42.4 (32.7)
t_1/2,_ h	–	4.9 (19.1)	7.3 (38.6)
V_z_/F, L	–	179.8 (34.9)	436.1 (47.6)

Data represent the arithmetic mean (percentage coefficient of variation) except for T_max_ and T_max,ss_, which are the median (range).

*AUC* area under the plasma concentration–time curve. *C*_*max*_ maximum plasma concentration. *T*_*max*_ time to reach C_max,_ *T*_*maxx,ss*_ time to reach C_max_ at steady state. *t*_*1/2*_ apparent terminal elimination half-life. *R*_*ac*_ accumulation ratio based on AUC_ss_, *CL*_*ss*_*/F* apparent oral clearance, *V*_*z*_*/F* apparent volume of distribution.

### Efficacy

**Fig. 1:**
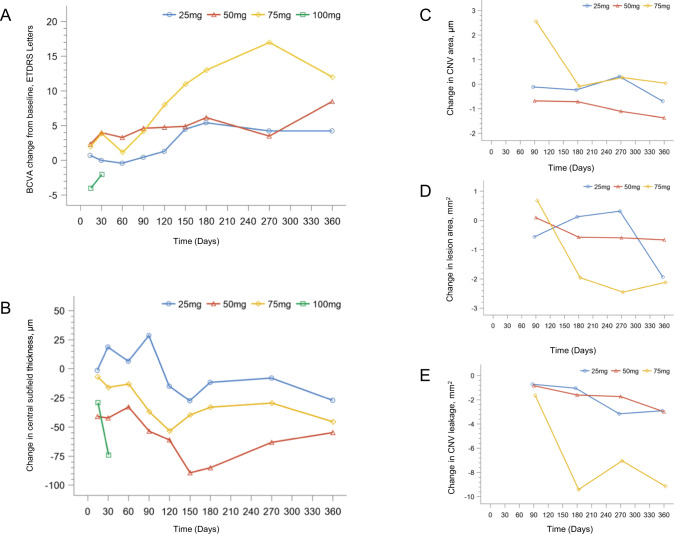
Change in best-corrected visual acuity (BCVA). **A** Central subfield thickness, (**B**) CNV area, (**C**) lesion area, (**D**) and CNV leakage, (**E**) from baseline to Day 360.

The mean BCVA changes in study eyes from baseline are shown in Table 4 and Fig. 1. Participants gained +6.4 letters (range, −14–27) by Day 180 and +7.7 letters (range, −5–29) by Day 360. Furthermore, 12 (29.3%) participants evaluable at Day 360 maintained or improved vision. In these 41 participants, mean decreases in CST (−44.0 μm; range, −258–40), CNV area (−0.9 mm^2^; range, −3.8–0.2), lesion area (−1.3 mm^2^; range, −4.8–0.5) and CNV leakage (−4.0 mm^2^; range, −10.8–0.2) were also observed (Fig. 1).

**Table 4:** Changes of best-corrected visual acuity and central subfield thickness.

Cohorts	*N*	Mean Change from Baseline to day 180		Mean Change from Baseline to day 360	
		BCVA (letters)	CST (μm)	BCVA (letters)	CST (μm)
25 mg	16	+5.4	−11.7	+4.3	−27.0
50 mg	16	+6.2	−85.0	+8.5	−54.8
75 mg	8	+13.0	−33.0	+12.0	−45.5
100 mg	1	NA	NA	NA	NA
All	41	+6.4	−48.6	+7.7	−44.0

*N* number of evaluable participants at Day 180 or Day 360, *BCVA* best-corrected visual acuity, *CST* central subfield thickness.

## Discussion

Preclinical studies have demonstrated that oral administration of vorolanib strongly repressed CNV [16]. In the present study, escalating doses of the VEGFR/PDGFR inhibitor vorolanib (25, 50, 75 and 100 mg) were administered to participants with nAMD. This is the first clinical trial to target VEGFR and PDGFR via oral therapy in participants with nAMD in China. Moreover, anatomical and visual improvements in participants with nAMD were achieved without any vorolanib-related ocular AEs.

In Caucasian participants with nAMD, ocular AEs occurred in 4 of 35 (11.4%) participants, although none were related to vorolanib [17]. In addition, 94 systemic AEs occurred, of which 32 were vorolanib-related [17]. There were no deaths or DLTs, and 4 serious AEs in 3 participants (8.6%) were recorded, but none were attributed to vorolanib [17]. Moreover, no MTD was observed in the study. Serious AEs occurred in three Chinese participants, all of whom had elevated transaminase levels (2 in the 25 mg cohort and 1 in the 100 mg cohort), including one 78-year-old participant with grade 2 increased alanine aminotransferase and aspartate aminotransferase, one 60-year-old participant with grade 3 increased alanine aminotransferase and aspartate aminotransferase, and one 67-year-old participant with grade 3 increased alanine aminotransferase and aspartate aminotransferase. All participants recovered after symptomatic treatment. In addition, drug-related AEs leading to drug discontinuation occurred in 6 of 41 participants (14.6%). No fatal adverse events were observed. No serious AEs related to vorolanib were reported in Caucasians; however, moderate-to-severe abnormal liver function tests were observed [17]. In addition, the different drug exposures between American and Chinese participants should also be noted. In phase 1 studies evaluating participants with solid tumours, the recommended dose of vorolanib monotherapy in US participants with advanced cancer is 400 mg once daily, while vorolanib showed an acceptable safety profile and preliminary activities over the dose range of 50–250 mg once daily in Chinese participants [18]. Moreover, the preliminary PK results show a relatively higher AUC in Chinese people than in Americans, which was possibly due to the lower weight of Chinese people compared with Americans (unpublished data). Overall, the most common TRAE was elevated alanine aminotransferase, indicating a favourable safety profile that was comparable with the results reported in the US [17].

The mean BCVA improved almost 6.4 letters on Day 180 after vorolanib treatment, which was better than that reported in the US study with a mean BCVA of +3.8 letters at 24 weeks [17]. On Day 180, participants in the 25, 50 and 75 mg cohorts gained 5.4, 6.2 and 13.0 letters from baseline, respectively. Additionally, the mean BCVA improved almost 7.7 letters at 360 days after vorolanib treatment. Moreover, the gains in BCVA seemed durable, with a mean BCVA improvement of 2.6 letters in 75.6% of participants who continued follow-up one month after vorolanib treatment. In addition, a significantly reduced CST was observed at 180 days and 360 days after oral vorolanib. By Day 180, the mean reduction in CST was 48.6 μm, which was comparable with participants reported in a US study that completed 24 weeks with a mean reduction in CST of 50 μm [17]. CNV leakage declined, which was reported in participants who received ranibizumab and bevacizumab [19, 20]. Moreover, the decline in CNV leakage was also accompanied by a reduced CNV area of 37.4%, which was comparable to that of patients treated with pegaptanib and ranibizumab [21, 22]. Despite continued CNV growth in participants with pegaptanib treatment, it grew slowly compared with that in patients who received a sham injection [21].

With continuous dose administration over the dose range of 50 to 75 mg, a steady-state concentration of vorolanib was reached after 4 days, and no obvious accumulation was observed, which was consistent with the relatively short half-life of vorolanib. Therefore, 25 mg dosing three times daily might also be appropriate.

This study has several limitations, such as a small sample size, no control group, high variability of BCVA measurement in eyes and different visual potentials. Compared with the intravitreous injection approach of anti-VEGF agents, oral administration can relieve the treatment burden, providing a more flexible and manageable administration for long-term outcomes.

In conclusion, TRAEs after oral administration of vorolanib up to a maximum dose of 100 mg were manageable in this phase 1 study. Given the limitations of the efficacy of anti-VEGF therapy and the burden of repeated intravitreal injections, alternate therapies are being explored. The desire to reduce injection frequency has promoted the development of sustained-release formulations of anti-VEGF drugs, as well as topical and oral formulations. Vorolanib, an oral tyrosine kinase inhibitor of both the VEGF and PDGF receptors, does provide important evidence that oral tyrosine kinase inhibitors have efficacy with manageable safety profiles in nAMD. However, we must acknowledge that the systemic adverse profile of vorolanib needs attention, and the potential benefits of vorolanib need to be carefully weighed against the risks. Considering that the pathogenesis of CNV formation in nAMD is complex and includes various factors that could be targeted in future treatments, emerging therapies, along with novel anti-VEGF therapies that address treatment burden (including safe oral treatments), are still warranted to provide the retina community with better options for managing nAMD.

## Summary

### What was known before

- Intravitreal anti-VEGF drugs (e.g., bevacizumab, ranibizumab, and aflibercept) have become the standard treatment for nAMD.
- Participants may require a high injection frequency over years of treatment, leading to a high treatment burden or several complications.
- The real-world studies showed worse visual outcomes, possibly due to poor compliance.
- Vorolanib is an oral VEGFR/PDGFR inhibitor that showed potential benefits in Caucasian participants with nAMD.

### What this study adds

- As an oral VEGFR/PDGFR inhibitor for nAMD, vorolanib was more convenient than intravitreal injection.
- This study reports the one-year efficacy and safety of oral vorolanib for nAMD.

### Supplementary information

Supplementary Table

## Data Availability

The datasets used and/or analysed during the current study are available from the corresponding author on reasonable request.
