# Supplementary material for: Safety and tolerability of oral vorolanib for neovascular (wet) age-related macular degeneration: a phase I, open-label study
Source: Eye (Lond). 2023 Apr 11;37(15):3228–33. doi: 10.1038/s41433-023-02496-x (PMC10564794; doi:10.1038/s41433-023-02496-x)
Supplement: Supplementary file 1 — Supplementary Table [file 41433_2023_2496_MOESM1_ESM.docx]

**Table S1** Schedule of procedures by visit

|  | **Screening** | **Baseline** | **PK** | **Continuous dosing** | | **Continuous dosing^※^** | | | | | **Extended phase**^11^ | **EOT visit**^11^ |
| --- | --- | --- | --- | --- | --- | --- | --- | --- | --- | --- | --- | --- |
| Visits | V1 | V2 | V3^5^ | V4^6^ | V5^7^ | V6 | V7 | V8 | V9 | V10 | V11+ |  |
|  | ≤-7 Days | Day 0 | Day 1/2 | Day 14 | Day 30 | Day 60 | Day 90 | Day 120 | Day 150 | Day 180 | Day 270^9^ | 30 Days after the last visit |
| **General Parameters** | | | | | | | | | | | | |
| Informed consent | X |  |  |  |  |  |  |  |  | X^10^ |  |  |
| Eligibility criteria | X |  |  |  |  |  |  |  |  |  |  |  |
| Medical history | X |  |  |  |  |  |  |  |  |  |  |  |
| Concomitant meds | X | (X) | X | X | X | X | X | X | X | X | X | X |
| Physical exam | X |  | X^2^ |  |  |  |  |  |  |  |  |  |
| Height and weight | X |  |  |  |  |  | X |  |  | X | X |  |
| Vital signs | X | (X) | X | X | X | X | X | X | X | X | X | X |
| Electrocardiogram | X |  | X^3^ | X^4^ | X^4^ |  | X^4^ |  |  | X^4^ | X |  |
| Administration of vorolanib |  |  | X | X | X | X | X | X | X | X | X |  |
| **Ophthalmic Parameters^@^** | | | | | | | | | | | | |
| Visual acuity (ETDRS) | X^&^ |  |  | X^&^ | X^&^ | X^&^ | X^&^ | X^&^ | X^&^ | X^&^ | X^&^ | X^&^ |
| Slit lamp exam | X |  |  | X | X | X | X | X | X | X | X | X |
| Intraocular pressure | X |  |  | X | X | X | X | X | X | X | X | X |
| Fundoscopy | X |  |  | X | X | X | X | X | X | X | X | X |
| Fluorescein angiography | X |  |  |  |  |  | X |  |  | X | X^8^ |  |
| Fundus photography | X^&^ |  |  |  |  |  | X^&^ |  |  | X^&^ | X^8^ |  |
| OCT | X^&^ |  |  | X^&^ | X^&^ | X^&^ | X^&^ | X^&^ | X^&^ | X^&^ | X^&^ | X^&^ |
| **Laboratory Parameters** | | | | | | | | | | | | |
| CBC w/differential | X |  |  | X | X | X | X | X | X | X | X | X |
| Biochemical exam | X |  |  | X | X | X | X | X | X | X | X | X |
| Urinalysis | X |  |  | X | X | X | X | X | X | X | X | X |
| Thyroid function (TSH, FT3, FT4) | X |  |  |  | X | X | X | X | X | X | X | X |
| Coagulation function | X |  |  | X | X |  | X |  |  | X | X | X |
| Lipase, amylase, creatine kinase | X |  |  | X | X |  | X |  |  | X | X | X |
| Blood pregnancy test | X |  |  |  |  |  |  |  |  |  |  |  |
| **Pharmacokinetic parameters** | | | | | | | | | | | | |
| Blood sample collection |  |  | X | X | X | X | X | X | X | X | X |  |
| **Safety** | | | | | | | | | | | | |
| Adverse events |  |  | X | X | X | X | X | X | X | X | X | X |

EOT, End of trial

^&^Ophthalmic examination of the contralateral eye.

^※^Whether the participants in four groups can continue to receive vorolanib should be comprehensively evaluated by the investigators.

^@^If there are abnormal laboratory indicators with clinical significance, the participants should be rechecked within one week.

^2^Physical examination is only performed on the first day before administration of vorolanib and within 2-6 h after administration of vorolanib.

^3^Electrocardiogram was performed at screening, pre-dose (-0.5 h) and 0.5, 2, 4, 8, 12, 24, 36 h post-dose at V3.

^4^Electrocardiogram was performed at each visit.

^5^The blood samples from participants in 25 mg group were collected at pre-dose, 0.5, 1, 2, 3, 4, 6, 8, 12, 16, 24, 36 and 48 h post-dose; the blood samples from participants in 50-100 mg groups were collected at pre-dose, 0.5, 1, 2, 3, 4, 6, 8, 12, 16, 24, 36, 48 and 72 h post-dose.

^6^The blood samples from participants in 25 mg group were collected at Day 14 and Day 30, while the blood samples from participants in 50 mg and 75 mg groups were collected at Day 7, Day 14 and Day 21.

^7^The blood samples from participants in 50 mg and 75 mg groups were collected at pre-dose, 0.5, 1, 2, 3, 4, 6, 8, 12, 16 and 24 h post-dose; then blood samples from participants only in 75 mg group were collected at 36, 48 and 72 h post-dose.

^8^Fluorescein angiography and fundus photography were performed at each visit during the extended phase.

^9^The visit in the extended period is employed every 90 days.

^10^Whether the participants were allowed for the extension phase was evaluated by investigator after the safety and efficacy evaluation. If the participant was suitable for the extension phase, the participant would be included in the extension phase after obtaining the informed consent.

^11^The administration period in this study of all participants was 1 year or the last participant received vorolanib for 6 months (whichever is shorter). Then the participants should be notified to complete the last visit within 5 days during the treatment period, and complete the EOT Visit within 30 days thereafter.
